# On the Comparative Value of Sulphuric Acid and Creosote in the Treatment of Alveolar Caries

**Published:** 1881-01

**Authors:** J. N. Farrar


### ON THE COMPARATIVE VALUE OF SULPHURIC ACID AND CREOSOTE IN THE TREATMENT OF ALVEOLAR CARIES.

BY J. N. FARRAR, M. D.

*Read before the Brooklyn Anatomical and Surgical Society.*

When or by whom creosote was first used in the treatment of alveolar caries I am not able to say; but as to sulphuric acid, as well as nitric acid, we find, according to Heister, who wrote in 1718, one hundred and sixty years ago, that it was then in use in the treatment of necrotic conditions of the jaw-bone. Since that time it has been more or less advocated, but with somewhat more interest after the report of Mr. Pollock's experiments in 1868–69, published in the *Lancet.*

For several years past, and until of late, the use of sulphuric acid, more especially in the form of elixir of vitriol, has been advocated by a few members of the dental profession, as the treatment *par excellence* not only for necrosis, but for alveolar abscess, the hypothesis set forth as positive theory by these gentlemen being that "acid would vigorously act to remove all the dead bone present without acting upon the surrounding living bone."

To test the real degree of virtue of aromatic sulphuric acid, I made in 1866–7, a long series of experiments with not only this form of acid preparation, but various degrees of aqueous solutions of all sorts of sulphuric acid, the commercial (always impure*),

* Sulphate of potassa, sulphate of lead, hyponitric acid, arsenic and chlorine are the usual impurities found even in the best commercial acids.

the Nordhausen, and the absolutely pure ordinary acid (prepared especially for my use). These experiments numbered about two hundred, made with great care, consuming almost all of my spare hours, days and nights, for over a year, and were not only made upon all conditions and forms of lifeless bone, but included vivisection experiments on sheep. The result showed that aromatic sulphuric acid has little or no effect for disintegrating bone of any sort, and that while dead bone long enough subjected to aqueous solutions will be affected by them, they act equally vigorously upon living bone; thus proving beyond doubt that however plausible and however desirable the hypothesis set forth, it had but very little scientific foundation.

Sulphuric acid is highly recommended by some persons for universal use in all conditions of abscess; but while it will frequently lead to fair results, and while it is undoubtedly to be preferred to creosote in cases of necrosis, I do not think its indiscriminate use in the treatment of simple abscesses, even though it be slightly antiseptic, is in accordance with the highest scientific principles.

Creosote has all the desired properties of sulphuric acid, which are indicated in the treatment of simple abscesses, with far superior antiseptic qualities; and as alveolar abscesses are not, as a rule, accompanied by necrosis proper, the former is superior for general use.

Even in the cases where acid is indicated, the final dressings, after the dead bone has been removed, may profitably be made with wood creosote, in order to secure its great antiseptic virtues, for the dead root is ultimately to be clothed like an encysted foreign body by the encroachment of the alveolar walls or the intermediate softer tissues, either by contracting around it, or by granulations or otherwise, thickened so as to embrace it.

Two varieties of creosote should be considered: escharotic and non-escharotic. Wood creosote is practically non-escharotic, and while this form may be superior for some cases where it is not desirable to destroy tissue, I think it is not equal to the other form in early stages of treatment, when destruction of the interior of the sac is indicated.

While creosote preparations are thus highly recommended for general abscess treatment, it is of the utmost importance that a correct diagnosis should be made. Should there be even slight necrosis present, the solution of the dead bone, so important to a final cure, will, for the time, be apt to be arrested by the use of creosote. I have known cases to be only apparently cured by this mistake. The disintegrative process and pus-formation for a time being arrested, and the fistula closed, there is produced what may be termed a "false cure," which remains only so long as the effect of the creosote prevents further decomposition of the dead tissue.

It is sometimes a nice point to determine whether there be necrosis present or not, when very limited in extent and difficult of access; but skilled examination will generally reveal by means of a delicate probe the difference between living and dead bone. Should there be, however, a doubt in the diagnosis, with strong suspicion of necrosis, it would appear to be a proper rule to give such cases the benefit of the doubt and treat with acid, the form and strength depending upon circumstances.

Having briefly considered the comparative virtues of the two kinds of creosote, let us confine our attention more especially to the acid aspect of our subject. Sulphuric acid — of which there are two varieties, the ordinary ($H_2SO_4$) and the Nordhausen ($H_2SO_4SO_3$) — in its pure state, has not to my knowledge been employed in practice; but the aromatic form known as "elixir of vitriol" has been in general use, as before stated. But in order to obtain a more vigorous action, various degrees of aqueous dilution of the pure acid have been thought to be more efficient in stubborn cases, where dead bone is associated. There are, however, some considerations respecting the acid treatment which, in my opinion, are of importance. Different and perhaps exaggerated views are entertained in regard to the relative value of the ordinary and the Nordhausen forms. This, however, only applies to the difference in degree of strength, and both may be used under the above modifications in aqueous solution; but in alcoholic (aromatic) solutions the Nordhausen is stronger, and acts more vigorously to destroy living soft tissues.

Chemical experiments upon dead tissue, outside of the living organism, as illustrative of similar conditions in the living, are always open to criticism, often absurd, especially in the direction of physiological chemistry. The chemical action of acids, even upon dead bone in and among living tissue, has been, and is by some people now, supposed to be somewhat different from their action upon outside dead matter.

How much and in what way so called "vitality" influences the action of chemical agents is not well known. I will not attempt to discuss the question. Many of these physiological queries are sometimes satisfactorily answered, but only on the basis of hypothesis. It is my belief that the difference in conditions of experiments is not so much a difference of kind of chemical action as a difference in the degree. We have dead and dying ossific tissue, and wish to prevent further extension of death, and get rid of the dead portion. What shall be done? Is there anything that will, when applied locally, hasten decomposition of the dead bone, and at the same time stimulate diseased living tissue to healthy action? Can any acid be made to dissolve it or not? And if so, will it dissolve the dead bone without acting similarly upon the living bone; and if so, can it be used in sufficient strength without injury to the surrounding soft tissues; and if so, to what degree and what preparations are best for practical use? Some of these questions I have already partially answered. Of course, in my remarks, it should always be borne in mind that systemic treatment, such as food and sunlight, have their influences in the formation of the "line of demarkation," as well as that of judicious general medical treatment.

Experiments show that necrosed bone may be acted upon, other things being equal, as well *within the jaw as without;* but in actual practice "other things" cannot be made equal; therefore allowances must be made—and great allowances, too. So far as the action of acids upon dead bone is concerned, we have the matter in a nutshell. Experiments show that necrosed bone can be dissolved by the use of some solutions of acid, but these experiments have led me to conclusions somewhat at variance with those generally held, especially in regard to the degree of value of the different forms of sulphuric acid. The unavoidable surrounding conditions in actual practice necessarily retard, if they do not interfere with, the action of chemical agents applied from without, even if an effort to retain them in place by the Heister method of the use of the tents of lint or cotton be made.

A sequestrum placed in a bottle containing an abundance of aqueous acid solution (although not at first) will, after from two to four hours, show decided chemical action upon it; but in practice, if we apply the same drug in the treatment of necrosis, the quantity must necessarily be very limited and also weak. The presence of detritus in and around the necrosed parts, and the natural secretions of the same, will so soon dilute and drive out this small quantity as to materially reduce the degree of chemical action.

These hindrances, added to the lack of time required to obtain full action of the acid in actual practice, would lead us to infer that if the soft tissues would tolerate it, a stronger solution would be necessary to accomplish the same degree of chemical action which a larger quantity of a weaker solution would do in the laboratory. A solution of about four parts of water to one of acid will not act upon necrosed tissue in the living jaw more than, if indeed as much as, a solution of about nine parts of water to one of acid on similar bone outside of the body, if kept at the temperature of the blood. Therefore, it would be natural to infer that to obtain more active results, the local application of acid in practice should be frequent. But the action of pure acid being very destructive to living tissue, we are obliged to use weaker forms in practice. But a strength of solution that will not destroy living tissue is so weak that, under the difficulties mentioned, it will not have so much disintegrative effect upon necrosed bone as at first might be supposed, unless the necrotic portion is sufficiently exposed to apply the acid directly upon it without coming in contact with living tissue — a condition of circumstances which probably would be better treated with a steel excavator. Yet there is no doubt that the frequent and long-continued use of some forms of solutions of acid will hasten somewhat the decomposition of dead bone, leading to an earlier cure; but whether this decomposition is from direct chemical action on the bone itself or through the increased activity of the vital process carried on by nature, is the question.

If we apply any of these solutions, even the aromatic sulphuric acid, to a case in practice where the necrotic portion can be observed, slight effervescence is noticed to take place for a short time. Now, in the natural decomposition of necrosed bone, the secretions are constantly holding in solution more or less of lime salts, and the carbonate coming in contact with sulphuric acid causes slight effervescence; but it does not extend much beyond the time necessary to change the lime in solution in the pus-detritus; this phenomenon is what has led to the erroneous supposition that it was demonstrative proof that the acid acts vigorously upon the bone itself.

Proof additional that the bone is not vigorously acted upon lies in the fact that, if we subject a piece of necrosed bone in the laboratory to pure aromatic sulphuric acid for three days and three nights, at 37.8° C., we will find, after re-weighing, that it has lost nothing; and while such bone, similarly treated with aqueous solutions of sulphuric acid of equal strength, will in the end be destroyed, the action is so slow at first that the change can only be seen after one or two hours. If this be so, it will readily be seen that, with all the hindrances mentioned in actual practice, we cannot depend much upon its direct chemical action upon bone itself, and upon close observation it will be found that the chief benefit from the acid treatment lies in the stimulation to the surrounding living parts which it effects, thus assisting Nature in her efforts to establish the line of demarkation, the formation of granulations behind the sequestrum leaving the dead bone in a condition to be more easily removed by instruments, and in cases where the sequestrum cannot be reached not preventing its natural decomposition, as would be the case if impregnated with creosote.

As to the comparative value of the aqueous solutions with the elixir of vitriol, circumstances must decide. While in most ordinary cases the aqueous solutions that will not destroy surrounding soft tissues must be so weak, there probably is but little if any difference. But where the bone is accessible, so that solutions may be applied without coming in contact with living tissues, stronger ones, even to the strength of one of acid to four of water, may be sometimes beneficial; but the action of sulphuric acid upon bone is so slow, that the more experience I have with it, the less faith I have in it as a disintegrator of necrosed bone.

